# Correlating symptoms and skin α-synuclein seeding parameters in olfactory dysfunction and Lewy body dementia

**DOI:** 10.1007/s00415-026-13745-w

**Published:** 2026-03-16

**Authors:** Oskar H. McWilliam, Remarh Bsoul, Aušrinė Areškevičiūtė, Ida S. B. Andersen, Marie Bruun, Christian von Buchwald, Steen G. Hasselbalch, Eva L. Lund, Christian K. Pedersen, Anja H. Simonsen, Gunhild Waldemar, Kasper Aanæs, Kristian S. Frederiksen

**Affiliations:** 1 Danish Dementia Research Centre, Department of Neurology, Copenhagen University Hospital–Rigshospitalet, Inge Lehmanns Vej 6, 2100 Copenhagen, Denmark; 2 Danish Reference Center for Prion Disease, Department of Pathology, Copenhagen University–Rigshospitalet, Copenhagen, Denmark; 3 Department of Clinical Medicine, Faculty of Health and Medical Sciences, University of Copenhagen, Copenhagen, Denmark; 4 Department of Otolaryngology, Head and Neck Surgery and Audiology, Copenhagen University Hospital–Rigshospitalet, Copenhagen, Denmark

**Keywords:** Seed amplification assay, Real-time quaking-induced conversion, Dementia with Lewy body, Hyposmia, Parkinson’s disease, Olfactory dysfunction

## Abstract

**Objective:**

We investigated profiles of clinical symptoms and kinetic α-synuclein seed amplification assay (αSyn-SAA) parameters in skin across the spectrum of Lewy body disease by comparing normosmic healthy controls (HC), individuals with idiopathic olfactory dysfunction (iOD), and dementia with Lewy bodies (DLB) patients.

**Methods:**

In this cross-sectional, case–control study, we included 49 αSyn-SAA-negative HC, 18 αSyn-SAA-positive iOD, and 25 αSyn-SAA-positive DLB patients. αSyn-SAA was performed on olfactory mucosa swabs and skin biopsies. Only the skin-derived kinetic parameters were explored for comparative kinetic analysis (13 iOD and 24 DLB). Clinical assessments included the Sniffin’ Sticks 16 for olfaction, Montreal Cognitive Assessment (MoCA), REM Sleep Behavior Disorder Single-Question (RBD1Q), and the Movement Disorder Society-Unified Parkinson’s Disease Rating Scale (MDS-UPDRS) I–III.

**Results:**

We observed a stepwise increase in symptoms across groups from HC to iOD to DLB, measured by MoCA (*p* < 0.05) and MDS-UPDRS I, II, III (*p* < 0.001). Participants with iOD and DLB did not differ in olfactory function (mean [SD]: 6.5 [2.4] vs. 6.0 [2.5], *p* = 0.51). Accelerated αSyn seeding kinetics, particularly shorter time to 50% of maximal fluorescence, were detected in DLB vs iOD (median hours [interquartile range] 17 [15–20] vs 14 [13–16], *p* = 0.017), achieving a classification accuracy of 74% (95% CI 57–91), and correlated with number of symptoms related to DLB without and with adjusting for age and sex (*p* = 0.002, *R*^2^ = 0.605).

**Conclusions:**

These findings delineate the Lewy body symptom profile in iOD positioning iOD as a prodromal stage in which peripheral αSyn aggregation kinetics can be detected.

**Supplementary Information:**

The online version contains supplementary material available at 10.1007/s00415-026-13745-w.

## Introduction

Manifest Lewy body diseases, such as Parkinson’s disease (PD) and dementia with Lewy bodies (DLB), are characterized by the pathological aggregation of α-synuclein (αSyn) within neurons, ultimately leading to neurodegeneration [1]. This aggregation process is thought to begin with a preclinical phase without symptoms, followed by a prolonged prodromal phase marked by a gradual emergence of symptoms, preceding the onset of manifest DLB or PD [2]. Idiopathic olfactory dysfunction (iOD) has been proposed as the earliest clinical precursor, occurring decades before parkinsonism and cognitive symptoms [3, 4]. Especially in DLB, it is an early and pronounced symptom, whereas in PD, olfactory dysfunction may be less pronounced early in the prodromal phase [3]. Although the phenotypes of PD and DLB may differ, the basic pathological mechanisms are believed to overlap [5], and shared prodromal features include REM sleep behavior disorder (RBD), psychiatric symptoms, daytime sleepiness, and autonomic dysfunction [3, 6–8]. However, current understanding of the prodromal stages of Lewy body disease remains limited and is largely based on models focusing on RBD, which is present in only approximately half of individuals with PD/DLB at the time of diagnosis [9]. Olfactory dysfunction, on the other hand, is present in more than 80% of de novo PD/DLB but may have a low specificity due to other etiologies affecting olfaction [9, 10].

The αSyn seed amplification assays (SAA) have been established as sensitive and highly specific methods for detecting pathological αSyn aggregates in various tissues, including cerebrospinal fluid (CSF), skin, and olfactory mucosa, and thus have the potential as an early diagnostic biomarker [11–13]. The αSyn-SAAs have primarily been interpreted as a binary outcome. However, αSyn-SAA holds promise for a more granular assessment of αSyn seeding properties, which may be more suited as a measure for tracking disease progression from the prodromal to the clinically manifest stage and may facilitate biomarker-based assessment of therapeutic response in future disease-modifying trials. However, establishing reliable continuous measurements has proven challenging. Poggiolini et al. [14] demonstrated in CSF that disease stage (RBD vs PD) and symptom clusters were correlated with kinetic parameters, including time-to-threshold, maximum slope (Vmax), and maximum fluorescence (Fmax). Similarly, using CSF-based αSyn-SAA, other studies [15, 16] reported an association between continuous measures and cognitive impairment and phenoconversion from prodromal to manifest Lewy body disease [17]. In skin αSyn-SAA, kinetic parameters have been investigated in PD and participants with RBD, with one study [18] reporting correlations with disease duration and severity, whereas another found no significant associations [19]. Kuzkina et al. reported that participants with RBD showed a faster seeding profile than those with PD in skin αSyn-SAA, possibly reflecting a more extensive peripheral distribution of pathological αSyn in the RBD phenotype [20]. However, to our knowledge, no studies have yet investigated kinetic αSyn-SAA parameters in minimally invasive peripheral tissues such as skin in a broad and early prodromal model like iOD versus manifest Lewy body disease.

This study aimed to further characterize the clinical symptom profile in αSyn-SAA positive iOD versus normosmic healthy controls (HC) and manifest Lewy body disease. Furthermore, we aimed to explore differences in kinetic αSyn-SAA parameters in skin across the disease stages of Lewy body disease. We also investigated the association between kinetic skin αSyn-SAA parameters and the severity of prodromal symptoms.

## Methods

### Study design

This is a cross-sectional, single-center, case–control study, which included HC, iOD, and DLB patients. A subset of findings from this cohort has previously been reported, focusing on the technical validation of the αSyn-SAA in HC and DLB [13] along with the discrimination between DLB and Alzheimer’s disease [21], as well as the dichotomous SAA outcomes in HC versus iOD [22]. The study was registered at clinicaltrials.gov (NCT05740683 + NCT05768425), and ethical approval was obtained by the Capital Region Ethical Committee (H-22053428 + H-22046053).

### Participants

**Fig. 1:**
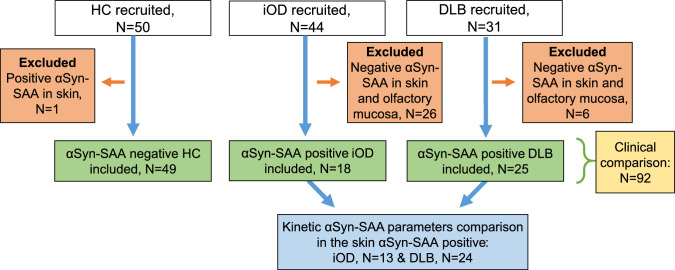
Inclusion flowchart. HCs were recruited from previous studies and through social media. The number of analyzed skin samples was: 49 HC, 42 iOD, and 31 DLB. The number of olfactory mucosa samples analyzed was: 50 HC, 43 iOD, and 30 DLB. One HC was excluded due to a positive αSyn-SAA result in skin. Among iOD, 26 were negative for αSyn-SAA in olfactory mucosa and/or skin and were therefore excluded. Of the 18 αSyn-SAA-positive iOD cases, five were positive in only olfactory mucosa or skin, while eight were positive in both biospecimens. In the DLB group, six patients were αSyn-SAA-negative in olfactory mucosa and/or skin and were excluded. Among the remaining DLB cases, one was positive only in the olfactory mucosa, 14 were positive only in the skin, and 10 were positive in both biospecimens. Clinical comparisons were performed between αSyn-SAA-negative HCs, αSyn-SAA-positive iOD, and αSyn-SAA-positive DLB. Analyses of αSyn-SAA kinetic parameters were restricted to participants with skin positivity and included 13 iOD and 24 DLB. *αSyn*-*SAA* α-Synuclein seed amplification assay, *DLB* dementia with Lewy bodies, *HC* healthy controls, *iOD* idiopathic olfactory dysfunction, *N* number

The inclusion criteria for HC and iOD were: (1) age between 55 and 75 years and (2) no major neurological disease (3) normal olfactory function (for HC) or idiopathic olfactory dysfunction (for iOD). Olfactory dysfunction was defined as Sniffin' Sticks identification test 16 (SIT16) ≤ 11 for participants aged 55–59 years and ≤ 10 for those aged 60 + years, as described in a previous study [22], see also Supplementary Materials S1 for further details. Participants with iOD were recruited from the Unit for Sense of Taste and Smell at Rigshospitalet or private practice ear, nose, and throat specialists (ENT) clinics (Fig. 1).

Participants diagnosed with probable DLB [23] or mild cognitive impairment with Lewy bodies [24] were recruited from the Memory Clinic at Copenhagen University Hospital, Rigshospitalet.

In this study, we included participants with DLB and iOD, who had positive αSyn-SAA in skin and/or olfactory mucosa, and HC with negative αSyn-SAA tests (Fig. 1).

### Clinical assessments

The clinical comparisons were done for all αSyn-SAA negative HC (*N* = 49), αSyn-SAA positive iOD (*N* = 18), αSyn-SAA positive DLB (*N* = 25). The olfactory function was assessed using a Danish adaptation of SIT16 [25], ranging from 0 to 16, with a lower score indicating more olfactory dysfunction. The Montreal Cognitive Assessment (MoCA) was used to assess cognitive function, and cognitive dysfunction was defined as < 26 points [26]. The REM Sleep Behavior Disorder Single-Question Screen (RBD1Q) was used to screen for possible RBD. The Movement Disorders Society-Unified Parkinson’s Disease Rating Scale (MDS-UPDRS) Parts I–III were applied to evaluate Lewy body symptoms. Part I assesses non-motor symptoms (e.g., subjective cognition, psychiatric, and dysautonomia symptoms), part II subjective motor symptoms, and part III objective motor performance [27]. Parkinsonism, neuropsychiatric symptoms, and autonomic dysfunctions were evaluated by subscores of the MDS-UPDRS (see Supplementary Materials S2 for further details). We constructed a composite symptom score by counting the presence (range: 0–8) of the following Lewy body symptoms: RBD, parkinsonism, cognitive impairment, orthostatic hypotension, constipation, urinary urgency, excessive daytime sleepiness, and depressed mood.

### Sampling and seed amplification assay

Two 3 mm skin punch biopsies were collected 5 cm paramedially from C7, as described previously [13]. The samples were kept on ice, dabbed for blood, rinsed with cold 5 mL phosphate-buffered saline, and stored dry at −80 °C within 2 h of sampling until the chemical and mechanical lysis [13]. Two skin samples were analyzed per participant in the iOD and HC groups, whereas only one sample was analyzed for participants with DLB. The weight-to-volume ratio was identical across all groups.

Olfactory mucosa samples were collected via endoscopic-guided swabbing with either nylon (FLOQswabs™ Copan® Diagnostics) or fiber-based swabs (Nasopharyngeal Specimen Collection Swab, NEST®), as previously described [13, 22]. Tubes with swabs and 3 mL saline were vortexed at a speed of 18,0000 rounds per minute for 1 min. The protein extraction for DLB was performed with mechanical lysis [13], whereas for HC and iOD it was performed with a method adopted from Bongianni et al. [28]. The olfactory mucosa samples were not analyzed for kinetic parameters due to the variations in swab type and protein extraction protocols between iOD/HC and DLB. The fluorescent development over time for iOD and HC is illustrated in Supplementary Fig. S1 and for DLB in the previously published article [13]. All blood-contaminated olfactory mucosa samples were excluded from αSyn-SAA.

αSyn-SAA was conducted uniformly by Real-Time Quaking-Induced Conversion with an in-house recombinant N-terminal 6x-histidine-tag α-synuclein substrate. The assay was performed on a FLUOStar™ Omega instrument for 48 h, with fluorescence measurement every 45 min as previously described [13]. αSyn-SAA was performed in quadruple wells and considered positive when ≥ 2 wells exceeded a preset threshold of 4 standard deviations (SD) above the thioflavin T baseline of the negative controls, as described previously [13, 29]. The same SAA setup has been validated in CSF against neuropathologically confirmed LBD [30], and SAA in skin, together with olfactory mucosa, has been evaluated against CSF in DLB [13].

### Kinetic seed amplification assay parameters

Kinetic αSyn-SAA analysis was performed on skin biopsies from all participants who tested positive for αSyn-SAA in skin tissue (13 iOD and 24 DLB) (Fig. 1). Based on previous studies of αSyn-SAA in CSF [14, 31], the following kinetic parameters were analyzed: (1) Maximum fluorescence (Fmax); (2) area under the curve (AUC); (3) time-to-threshold (sometimes referenced as lag time); (4) TH50, the time in hours to reach 50% of Fmax; (5) maximum slope (Vmax), calculated as the highest rate of fluorescence increase across two time points. We also calculated the quenching ratio, which was defined as Fmax divided by the last fluorescent value. See Supplementary Materials Fig. S2 for illustrated definitions of kinetic parameters.

αSyn-SAA for iOD/HC samples was conducted using a single batch of recombinant αSyn, and the DLB samples were tested with two different recombinant αSyn batches. Different brain homogenate dilutions from a single DLB patient were made uniformly and used freshly as the positive controls in both DLB and iOD/HC αSyn-SAAs. The batch used for iOD/HC demonstrated more rapid kinetics, e.g., shorter TH50 and higher Fmax and AUC than those used for DLB (Supplementary Materials Fig. S3), which performed alike.

### Statistical analysis

The statistical software R with RStudio (version 4.3.0) was used for all statistical analyses. Comparisons between HC, iOD, and DLB were conducted using either ANOVA or independent-samples t-tests for normally distributed variables, and Kruskal–Wallis or Wilcoxon rank-sum tests for nonparametric data. Chi-squared (N > 5) or Fisher’s exact test (N ≤ 5) was employed for categorical data comparisons. *p* values for clinical assessments were corrected post hoc pairwise with Holm’s method. Associations between clinical symptoms were assessed using Spearman’s rank correlation. Linear regression models were employed to evaluate the relationship between SAA kinetic parameters as predictors and clinical assessments as outcomes, with and without adjusting for age and sex as covariates. The accuracy of each parameter in distinguishing between iOD and DLB was calculated using the pROC package with Youden’s Index. The robustness was tested using Leave-One-Out Cross-Validation, and the SD was calculated with bootstrapping.

## Results

One HC was αSyn-SAA positive in skin and thus excluded from analysis. A total of 18 participants with iOD were αSyn-SAA positive in olfactory mucosa (*N* = 13) and/or skin (*N* = 13), while 25 DLB patients were positive in olfactory mucosa (*N* = 11) and/or skin (*N* = 24) (Fig. 1). No significant clinical differences were observed between iOD positive only olfactory mucosa versus iOD positive in both biospecimens or only in skin (data not shown). All skin αSyn-SAA positive DLB cases, and all but one iOD case, showed positive seeding activity in all four assay wells. Participants with DLB were older than HCs and individuals with iOD (Table 1), were more often males, and reported a longer duration of subjective olfactory dysfunction compared to iOD. Possible RBD and medication for autonomic dysfunction were more common in DLB, but not significantly different between the HC and iOD groups.

**Table 1:** Demographics and clinical variables

	HC (*N* = 49)	iOD (*N* = 18)	DLB (*N* = 25)	*p* value (HC vs iOD)	*p* value (iOD vs DLB)	*p* value (all)
Sex, *N* female (%)	27 (55%)	11 (61%)	3 (12%)	0.66	0.003	< 0.001
Age, mean years (SD)	68 (5.1)	68 (4.3)	75 (5.1)	0.72	< 0.001	< 0.001
Never smoker, *N* (%)	21 (43%)	8 (44%)	10 (40%)	0.52	0.84	0.95
Depression (current or previously), *N* (%)	5 (10%)	6 (33%)	4 (16%)	0.006	0.15	0.028
Hypertension, *N* (%)	21 (43%)	5 (28%)	13 (52%)	0.26	0.15	0.31
Olfactory dysfunction onset, *N* (%)						
≤ 1 year	NA	2 (11%)	1 (5%)	NA	0.016	NA
> 1–2 years	NA	1 (6%)	2 (10%)			
> 2–5 years	NA	7 (39%)	4 (20%)			
> 5–10 years	NA	6 (33%)	1 (5%)			
≥ 10 years	NA	2 (11%)	12 (60%)			
Use of laxatives, *N* (%)	5 (10%)	4 (22%)	13 (52%)	0.096	0.13	< 0.001
Use of medication for urinary urge, *N* (%)	1 (2%)	0	12 (48%)	> 0.99	0.003	< 0.001

Demographics in HC, iOD, and DLB. Olfactory dysfunction onset was defined as the self-reported time at which the individual first noticed a reduced sense of smell. No clinical differences were found between iOD only αSyn-SAA positive in olfactory mucosa (*N* = 5) versus iOD αSyn-SAA positive in skin (*N* = 13)

*DLB* dementia with Lewy bodies, *HC* healthy controls, *iOD* idiopathic olfactory dysfunction, *N* number, *NA* not applicable, *SD* standard deviation

**Fig. 2:**
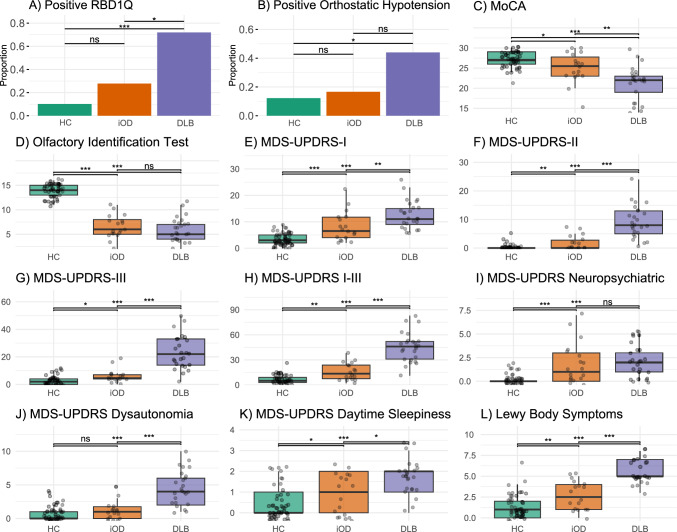
Clinical assessments and symptoms. Clinical assessments across groups (HC, iOD, and DLB). The *p* values are corrected pairwise with Holm’s test. *p ≤ 0.05, **p < 0.01, ***p ≤ 0.001, ****p ≤ 0.0001. *ns* nonsignificant, *DLB* dementia with Lewy bodies, *HC* healthy controls, *iOD* idiopathic olfactory dysfunction, *MDS*-*UPDRS* Movement disorders society unified, *MoCA* Montreal cognitive assessment, *RBD1Q* REM sleep behavior disorder single question

As expected, symptoms were most prominent in participants with DLB and least pronounced in HCs (Table 1 and Fig. 2), whereas the results for iODs tended to be intermediate between DLB and HCs. No significant differences were observed between the iOD and DLB groups in measures of current olfactory function or neuropsychiatric symptoms (Fig. 2B, I). Furthermore, symptoms of autonomic dysfunction did not differ between the iOD and HC groups (Fig. 2J).

Olfactory and autonomic dysfunction were negatively correlated within the iOD and DLB groups (Supplementary Fig. S4A, B), whereas the duration of olfactory dysfunction and the degree of cognitive impairment correlated positively (Supplementary Fig. S4A). In the pooled iOD and DLB group, autonomic dysfunction was positively associated with increased daytime sleepiness, parkinsonian motor symptoms, cognitive impairment, and RBD (Supplementary Fig. S4C).

**Fig. 3:**
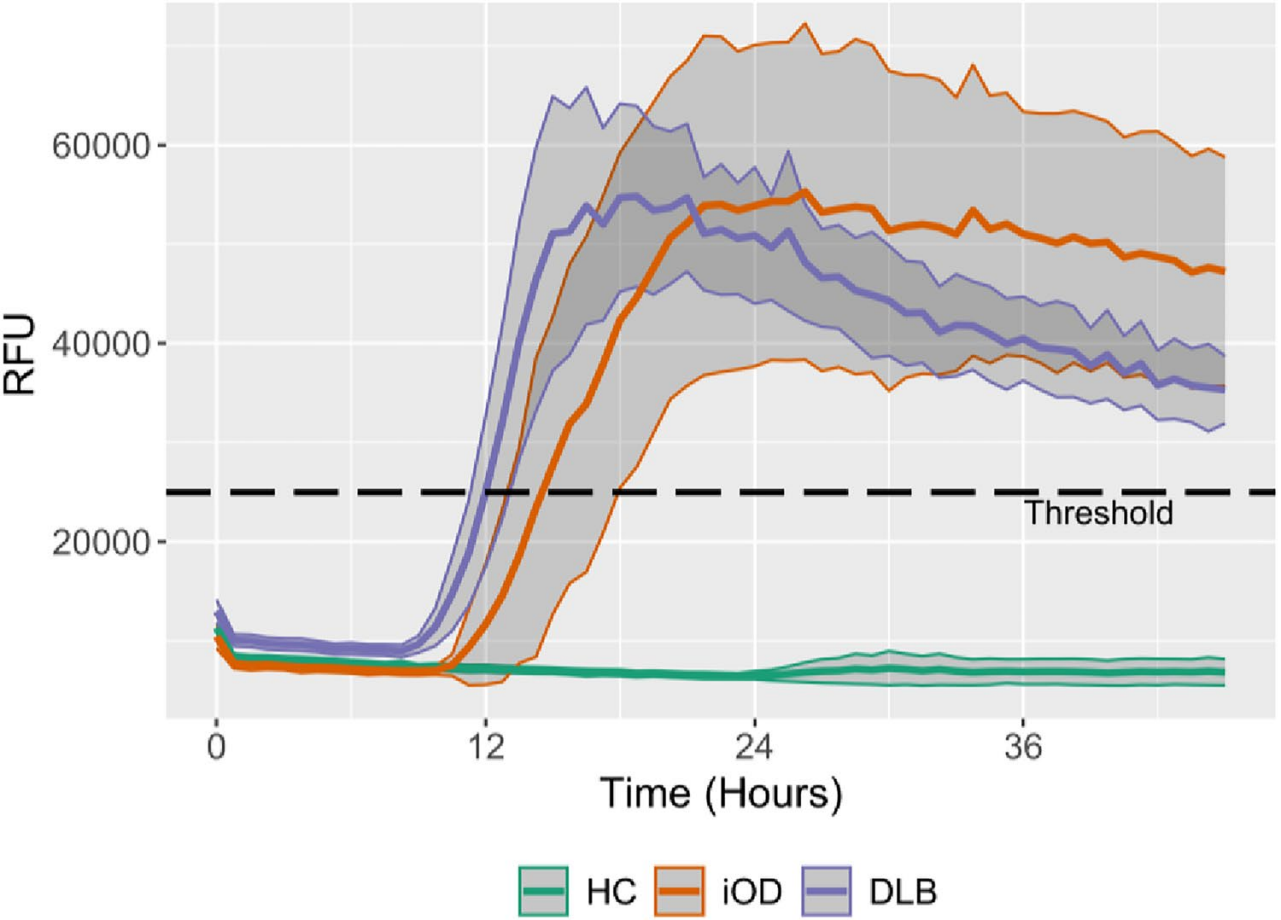
α-Synuclein seeding in skin over time. Time plot of the seed amplification from skin sample analysis with mean and CI for each group: HC (*N* = 48 αSyn-SAA negative in skin), iOD (*N* = 13 αSyn-SAA positive in skin), and DLB (*N* = 24 αSyn-SAA positive in skin). To assess the maximal seed potential, the plot is based on the mean values from the two best-performing wells out of four, as described previously [13]. Single participant data can be viewed in the Supplementary Materials Fig. S5. *DLB* Dementia with Lewy bodies, *CI* 95% confidence intervals, *HC* healthy controls, *iOD* idiopathic olfactory dysfunction, *RFU* relative fluorescence unit

The kinetic parameters from αSyn-SAA in skin from HC and the αSyn positive iOD and DLB are displayed in Fig. 3 and Table 2. In these assays, the DLB group exhibited faster seeding kinetics compared to the iOD group, as indicated by shorter TH50 and time-to-threshold values. The AUC did not differ between iOD and DLB but was nominally higher in DLB (iOD vs DLB median (IQR) 2.4 (1.6–2.6) × 10^6^ vs 3.2 (1.7–4.6) × 10^6^, *p* = 0.11) when corrected for the quenching ratio. DLB and iOD cases that were RBD1Q-positive showed a nominal but non-significant faster seeding compared with RBD1Q-negative cases (Supplementary Table S1).

**Table 2:** Kinetic parameters for the α-synuclein seeding in skin samples

	iOD (*N* = 13)	DLB (*N* = 24)	*p* value
Time-to-threshold, median, (IQR), hours	16 (14–19)	13 (11–17)	0.029
TH50, median (IQR) hours	17 (15–20)	14 (13–16)	0.017
Vmax, median (IQR) × 10^3^ RFU	11 (8.8–17)	14 (8.9–26)	0.31
Fmax, median (IQR) × 10^3^ RFU	66 (41–81)	65 (50–96)	0.63
AUC, median (IQR) × 10⁶	1.6 (1.1–1.9)	1.6 (1.3–1.7)	0.91
Quenching ratio, median (IQR)	1.4 (1.3–1.4)	2.1 (1.5–2.2)	0.0015

Kinetic parameters for skin SAA in SAA-positive iOD and DLB. See also Supplementary Materials Fig. S2 for illustrated definitions of kinetic parameters

*AUC* area under the curve, *DLB* Dementia with Lewy bodies, *Fmax* maximal fluorescence measured, *HC* healthy controls, *iOD* idiopathic olfactory dysfunction, *IQR* interquartile range, *RFU* relative fluorescence unit, *TH50* time to 50 of Fmax, *Vmax* maximal velocity of RFU change over 1.5 h

TH50 demonstrated the highest classification accuracy (DLB vs. iOD) at 72% (95% confidence interval (CI) 55–89) (Table 3), which remained robust under cross-validation with an accuracy of 70% (SD 7.4%). Time-to-threshold yielded a comparable accuracy to TH50, whereas Vmax, Fmax, and the fluorescence AUC all showed accuracies with confidence intervals overlapping 50%, indicating limited discriminative power.

**Table 3:** Accuracy, sensitivity, and specificity of kinetic parameters for iOD vs DLB

	Accuracy% (CI)	Sensitivity% (CI)	Specificity% (CI)	Threshold
Time-to-threshold	72 (55–89)	54 (33–75)	84 (65–100)	14 h
TH50	74 (57–91)	71 (50–88)	69 (46–92)	15 h
Vmax	61 (42–79)	42 (25–63)	92 (76–100)	17 × 10^3^ RFU
Fmax	55 (35–75)	88 (71–100)	39 (15–69)	80 × 10^3^ RFU
AUC	51 (29–73)	63 (48–83)	62 (38–85)	1.6 × 10⁶ RFU

Diagnostic performance of kinetic parameters for discrimination of DLB (case) from iOD (control). The accuracy, sensitivity, and specificity were calculated from ROC curves. See Supplementary Materials Fig. S6

*AUC* area under the curve (of the fluorescent activity), *CI* 95% confidence interval, *Fmax* maximal fluorescence measured, *RFU* relative fluorescence unit, *TH50* time to 50 of Fmax, *Vmax* maximal velocity of RFU change over 1.5 h

**Fig. 4:**
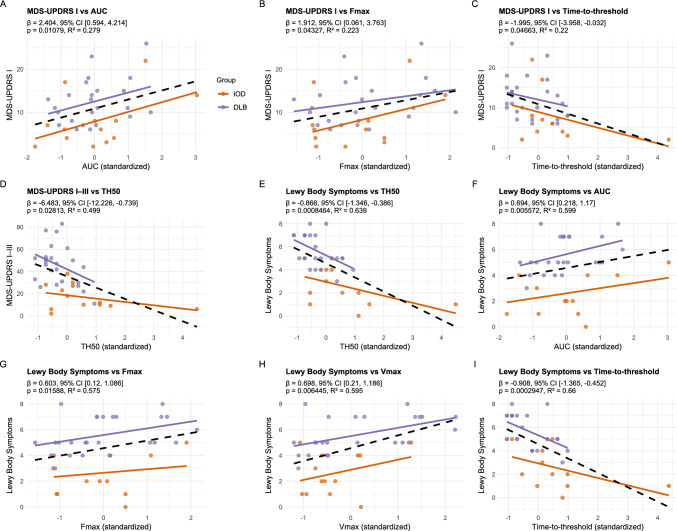
Association of symptoms and kinetic seeding parameters. Linear regression models between clinical assessment scale scores (outcome) and αSyn-SAA kinetic parameters (predictors) in skin samples corrected for age and sex. Orange line = iOD, purple line = DLB, and dashed line represents the combined group (iOD and DLB). Only significant associations are displayed. Full tables of uncorrected and corrected models can be viewed in the Supplementary Materials Tables S2 and S3. *AUC* area under the curve, *DLB* Dementia with Lewy bodies, *Fmax* maximal fluorescence measured, *HC* healthy controls, *iOD* idiopathic olfactory dysfunction, *TH50* time to 50 of Fmax, *Vmax* maximal velocity of RFU change over 1.5 h

Significant associations between kinetic parameters and symptom burden are illustrated in Fig. 4 (and Supplementary Tables S2 and S3). The total number of symptoms showed the strongest associations across kinetic measures, yielding the highest R^2^ values. In contrast, cognitive dysfunction and olfactory dysfunction were not significantly associated with kinetic parameters.

## Discussion

This study investigated clinical symptoms and explored αSyn seeding kinetic parameters in skin across the disease stages of Lewy body disease. We found increasing severity and frequency of several Lewy body symptoms across groups, from HC over iOD to DLB. This was paralleled by accelerated αSyn seeding parameters across groups and was associated with the severity of clinical symptoms. These findings are consistent with a model in which iOD represents a prodromal stage of Lewy body disease, characterized by neuropsychiatric symptoms, cognitive dysfunction, sleep disorders, parkinsonism, but not autonomic dysfunction, and peripheral αSyn aggregation may be detected and potentially correlate with symptom severity.

### Lewy body symptoms

The increasing severity and frequency of symptoms associated with Lewy body disease, such as motor parkinsonism, cognitive dysfunction, and daytime sleepiness, across HC, iOD, and DLB, indicate early onset and continuous symptom escalation over time, as would be expected. In the iOD group, cognitive dysfunction was associated with the duration of olfactory impairment (Supplementary Materials Fig. S3). Similar observations correlating olfaction and cognitive dysfunction were observed by Weintraub et al. [32], who found that performance on specific cognitive subtests correlated with disease progression in participants with olfactory dysfunction.

No significant differences were observed between the iOD and DLB groups in terms of olfactory dysfunction and neuropsychiatric symptoms such as anxiety, depression, and apathy (Fig. 2D, I). This finding suggests that these symptoms may already reach a level of severity comparable to that seen in manifest Lewy body disease during the earlier, prodromal stage. The early involvement of olfaction is further supported by findings in participants with RBD, where olfactory deficits are frequently observed decades before phenoconversion to manifest Lewy body disease. For example, Fereshtehnejad et al. [3] reported that olfactory dysfunction may already be present in participants with RBD more than 20 years before phenoconversion, with only a moderate, nonsignificant decline until conversion to DLB. The PD converters, on the other hand, had a more steep decrease in olfactory function within the last 10 years before phenoconversion. Participants converting to multiple system atrophy rarely have prodromal olfactory dysfunction [33], and thus are not likely to be part of the iOD group.

Higher levels of depressive symptoms have previously been reported in iOD versus HC [34, 35]. Our findings support these observations; however, we did not find more severe neuropsychiatric symptoms in DLB vs iOD. This may indicate a plateau of neuropsychiatric symptoms in the prodromal iOD phase.

Symptoms and treatment of autonomic dysfunction were more prevalent and pronounced in DLB compared to HC. In contrast, the iOD group, compared to HC, showed only a slight numeric but nonsignificant increase in the use of laxatives (Table 1), orthostatic hypotension, and dysautonomia score (Fig. 2B, J). This indicates that iOD may not be burdened with autonomic dysfunction to the same degree as other prodromal models, e.g., RBD.

### Kinetic seed amplification

To our knowledge, this is the first study to find significant differences in kinetic αSyn-SAA parameters in skin between iOD and DLB. Furthermore, we showed that some kinetic αSyn-SAA parameters correlated with the clinical symptom burden, particularly the number of Lewy body symptoms, as well as nonmotor symptoms (MDS-UPDRS I). Specifically, time-to-threshold and TH50 discriminated iOD from DLB with accuracies of 72% (CI 55–89) and 74% (CI 57–91), respectively. These findings are in line with previous reports showing that time-to-threshold and T50 in skin αSyn-SAA correlate with disease duration and severity in PD [18]. Also in CSF, the kinetic parameters correlated with disease stage, particularly in participants with RBD compared to those with PD [14]. However, results across studies are conflicting. One CSF-based study did not observe faster seeding in PD compared with prodromal Lewy body disease groups (RBD and iOD) [17]. Furthermore, Kuzkina et al. found faster seeding in participants with RBD compared with PD, suggesting that the seeding kinetics, besides stage, may also be phenotype/symptom dependent. This difference was explained by earlier αSyn accumulation in skin from the participants with RBD or a decrease in pathological αSyn due to nerve fiber loss, as suggested in some papers [36, 37]. We also observed a nominal trend toward faster seeding in the DLB and iOD with possible RBD compared with participants without RBD; however, this difference did not reach statistical significance (Supplementary Table S1). The faster seeding observed in DLB compared with iOD may reflect a higher burden of skin αSyn pathology [38] resulting from earlier peripheral involvement and more advanced disease stage. Alternatively, or in addition, differences in the strains of pathological αSyn between DLB and iOD may influence amplification kinetics. The connection between Lewy body disease stage and amount of αSyn pathology was corroborated by correlations between the severity of several clinical measures and kinetic parameters derived from αSyn-SAA within and across groups (Fig. 4, Supplementary Materials Tables S2 + S3). Notably, the number of Lewy body symptoms and nonmotor symptoms showed significant correlations with kinetic αSyn-SAA parameters, even after adjusting for age and sex. In contrast to previous studies using CSF-based SAAs [15, 39], we did not detect a correlation between cognitive performance (MoCA) and kinetic parameters.

A key challenge in interpreting our kinetic αSyn-SAA results was the markedly faster and stronger seeding observed in positive controls made from DLB brain homogenate, with different batches of recombinant α-synuclein used for iOD and DLB. However, without the batch-related variation, the differences between DLB and iOD seeding kinetics in skin would likely have been even greater. Furthermore, the proximity of the results and the robust correlations with clinical parameters within and across the groups support the assay’s validity (Fig. 4).

The findings indicate that kinetic αSyn-SAA parameters derived from a minimally invasive biospecimen are associated with both disease stage and clinical symptom burden, suggesting that skin αSyn-SAA may have potential utility as a stage-sensitive biomarker in Lewy body disease. These findings are explorative and should be validated in independent cohorts and larger samples with different prodromal populations, including longitudinal follow-up.

In HC, no olfactory and only one skin αSyn-SAA was positive, contrasting with previous reports of approximately 10% positivity [18, 37]. This likely reflects the inclusion of normosmic HCs, emphasizing the possibility of excluding control participants with olfactory dysfunction from cohorts to reduce the risk of incidental Lewy body disease.

### Limitations

In addition to the between-batch differences in positive controls noted above, this study has several limitations. Most notably, the limited sample size of αSyn-SAA-positive iOD participants warrants caution when interpreting the results, which are exploratory and need validation in independent cohorts. Furthermore, the cross-sectional design and lack of longitudinal follow-up restrict our ability to assess the progression of symptoms and αSyn-SAA kinetics over time. We have compared iOD with DLB and not PD participants. It would have strengthened the study to have included PD, as some participants with iOD (and perhaps even the majority of those who progress) will likely phenoconvert to PD rather than DLB. Nonetheless, olfactory dysfunction is an earlier and prominent prodromal symptom in DLB converters compared with PD, and there is a substantial clinical overlap between the two subtypes of Lewy body disease [3]. Although our study did not investigate different pathological αSyn strains, previous research has shown minimal differences in seeding activity between PD and DLB when adjusted for disease severity [40, 41]. In contrast, multiple system atrophy exhibits a distinct pathological αSyn strain with a markedly different seeding activity [42, 43], but as they do not exhibit olfactory dysfunction to the same degree as DLB and PD, this is unlikely to have influenced our findings.

For the analysis of clinical symptoms, we included iOD and DLB participants who were αSyn-SAA-positive in either the olfactory mucosa or skin, to increase the statistical power and reflect the hypothesis of distinct entry sites for pathological αSyn. iOD participants who were SAA-negative in skin but positive in the olfactory mucosa were excluded from the analyses, along with HCs, as many kinetic parameters cannot be calculated for samples that fail to reach the amplification threshold.

Our assessment of Lewy body symptoms relied mainly on clinical evaluations without the use of auxiliary investigations, such as imaging of dopaminergic function, autonomic dysfunction, or polysomnography, which could be more sensitive and precise in early stages. Moreover, diagnoses were based on clinical criteria without neuropathological confirmation. Nevertheless, the iOD diagnoses were made in a specialized unit for smell and taste disorders, and DLB diagnoses were determined through a multidisciplinary diagnostic process. Combined with the high specificity and sensitivity of αSyn-SAAs, this provides confidence in the diagnostic classifications [44].

As expected, the DLB group was older and had a higher proportion of male participants compared to the iOD and HC groups. A male predominance among patients with DLB has been reported in several studies [45]. While the demographic differences may have influenced certain clinical measures, we adjusted our linear models for age and sex and still observed significant correlations between Lewy body symptom burden and kinetic αSyn-SAA parameters.

### Strengths

This study employed a comprehensive set of clinical assessments to capture the spectrum of Lewy body symptoms. Procedures such as olfactory testing, nasal swabbing, and skin biopsy are minimally invasive, time-efficient, and can be performed without the need for prior auxiliary testing. The SAA kinetic analyses on skin and olfactory mucosa were conducted using a uniform protocol, enhancing both feasibility and translational potential.

## Conclusion

This study reinforces the role of iOD as a valuable prodromal model for Lewy body disease, and contributes to the characterization of iOD, which may differ from the RBD and pure autonomic failure phenotypes. Our findings contribute to the potential of αSyn-SAA as an early biomarker of Lewy body disease and broaden the understanding of the prodromal stage, complementing existing research focused primarily on RBD. Moreover, we show that faster seeding is associated with higher symptom burden in iOD and DLB. These findings should be validated in larger longitudinal cohorts, as they may in the future offer a powerful, minimally invasive tool for monitoring disease progression and evaluating treatment response to anti-α-synuclein therapies.

## Supplementary Information

Below is the link to the electronic supplementary material.

Supplementary file1 (DOCX 1428 KB)

## Data Availability

The data that support the findings of this study are available from the corresponding author upon reasonable request.
