# Supplementary material for: Correlating symptoms and skin α-synuclein seeding parameters in olfactory dysfunction and Lewy body dementia
Source: J Neurol. 2026 Mar 16;273(4):210. doi: 10.1007/s00415-026-13745-w (PMC12992478; doi:10.1007/s00415-026-13745-w)
Supplement: Supplementary file 1 — Supplementary file1 (DOCX 1428 KB) [file 415_2026_13745_MOESM1_ESM.docx]

# Supplementary Materials

Correlating Symptoms & Skin α-Synuclein Seeding Parameters in Olfactory Dysfunction & Lewy Body Dementia
Oskar H. McWilliam MD^1#^; Remarh Bsoul Msc^2^, Aušrinė Areškevičiūtė, MSc, PhD^2^, Ida SB Andersen^4^; Marie Bruun, MD, PhD^1,3^; Christian von Buchwald , MD, DMSc^3,4^; Steen G. Hasselbalch , MD, DMSc^1,3^; Eva L. Lund , MD, PhD^2,3^; Christian K. Pedersen, MD^4^; Anja H. Simonsen, MSc, PhD^1^; Gunhild Waldemar, MD, DMSc^1,3^; Kasper Aanæs, MD, PhD^3,4^; and Kristian S. Frederiksen, MD, PhD^1,3^

1) Danish Dementia Research Centre, Dept. of Neurology, Copenhagen University Hospital - Rigshospitalet, Copenhagen, Denmark
2) Danish Reference Center for Prion Disease, Dept. of Pathology, Copenhagen University Hospital - Rigshospitalet, Copenhagen, Denmark
3) Department of Clinical Medicine, Faculty of Health and Medical Sciences, University of Copenhagen, Copenhagen, Denmark
4) Dept. of Otolaryngology, Head & Neck Surgery and Audiology, Copenhagen University Hospital - Rigshospitalet, Copenhagen, Denmark

## S1 Definition of iOD

Criteria for iOD: a) non-congenital olfactory dysfunction with no temporal link to infections (within 2 weeks) or head trauma, b) magnetic resonance or computed tomography to exclude pathology relating to olfactory dysfunction or progressive olfactory dysfunction >2 years, c) no significant sino-nasal disease on endoscopy, and e) no response to short-term systemic/topical corticosteroid treatment. The exclusion criteria for both iOD and HC: a) a diagnosis of PD/DLB, b) a diagnosis of other major neurological/psychiatric diseases, or c) substance or alcohol abuse.

## S2 Definition of Parkinsonism, Neuropsychiatric Symptoms, and Autonomic Dysfunction

The neuropsychiatric score was calculated as the sum of values from the MDS-UPDRS questions on depression, anxiety, and apathy. The dysautonomia score was calculated as the sum of values from the MDS-UPDRS questions on urinary problems, constipation, lightheadedness on standing, and drooling. Parkinsonism was defined as MDS-UPDRS III score without action tremor >6.

## Figure S1 Seeding over Time in Olfactory Mucosa with individual datapoints


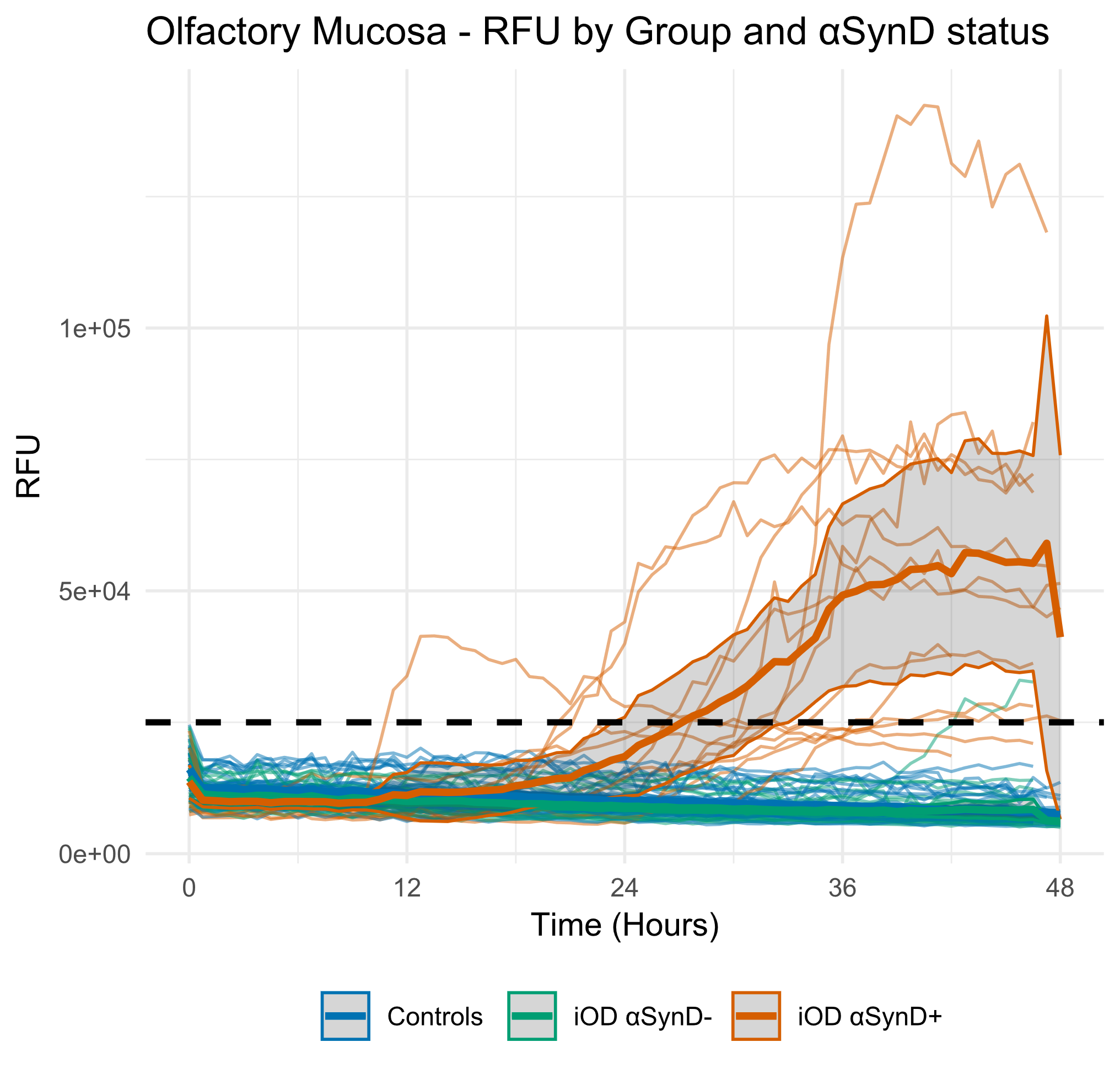


HC

iOD SAA-

iOD SAA+

*Time plot with individual datapoints of the seed amplification from olfactory mucosa sample analysis with mean and CI for each group: HC (N=49) and iOD (N=43). The plot is based on the mean values from the two best-performing wells out of four for the positive samples and for all four for the negative samples. The SAA data for DLB and HC has been previously published [13].
Abbreviations: DLB = Dementia with Lewy bodies, HC = healthy controls, iOD = idiopathic olfactory dysfunction, RFU = relative fluorescence unit, SAA = seed amplification assay*

## Figure S2 SAA Kinetics Parameters Definitions


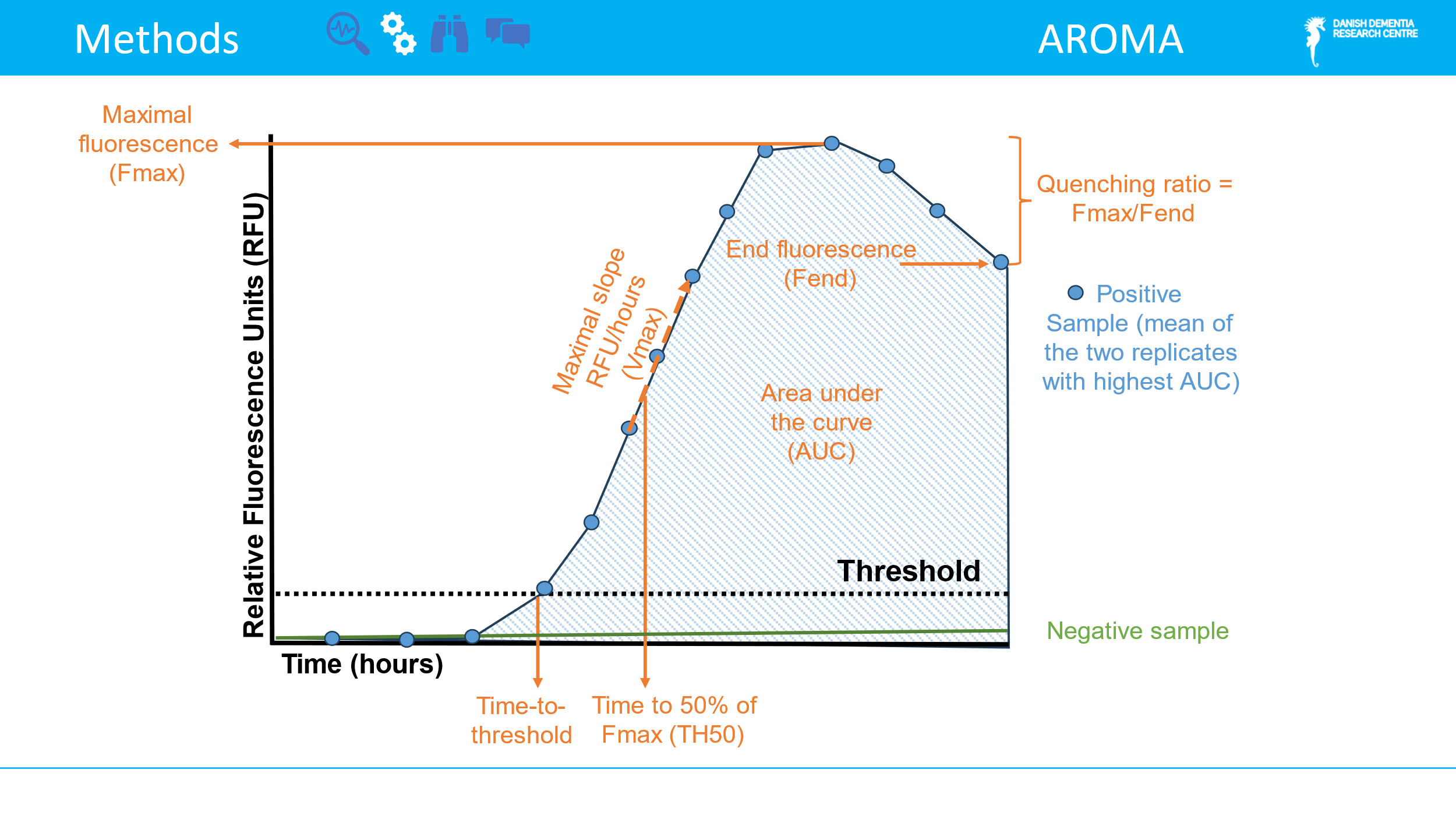


*Maximum fluorescence (Fmax), representing the peak intensity of the relative fluorescence units (RFU) signal; (2) area under the curve (AUC), reflecting cumulative fluorescence over time; (3) time-to-threshold (sometimes referenced to as lag time), defined as the time from 0 to when the fluorescence reached our threshold of 25,000 RFU, based on 4 SDs from a negative control; (4) TH50, the time in hours to reach 50% of Fmax; (5) maximum slope (Vmax), calculated as the highest rate of fluorescence increase across two time points (1.5 hours apart), to adjust for fluctuations in the fluorescence; and 6) the quenching ratio was defined as Fmax divided by the last fluorescent value.*

## Figure S3 SAA Kinetics in Different Recombinant α-Synuclein Batches

|  | Batch 1 iOD/HC (N=24) | Batch 2+3 DLB (N=50) | p-value |
| --- | --- | --- | --- |
| **TH50**, median (IQR) hours | 14  (13–15) | 23  (20–26) | **<0.0001** |
| **Time-to-threshold**, median (IQR) hours | 13  (11–14) | 23  (21–26.2) | **<0.0001** |
| **Vmax**, median (IQR)×10³ RFU | 16  (15–19) | 7.6  (5.8–10) | **<0.0001** |
| **AUC**, median (IQR)×10⁶ | 2.1  (2.0–2.1) | 0.94  (0.87–1.0) | **<0.0001** |
| **Fmax**, median (IQR)×10³ RFU | 82  (80–85) | 46  (42–55) | **<0.0001** |
| **Quenching Ratio**, median (IQR) | 1.4  (1.3–1.4) | 1.3  (1.2–1.4) | 0.23 |


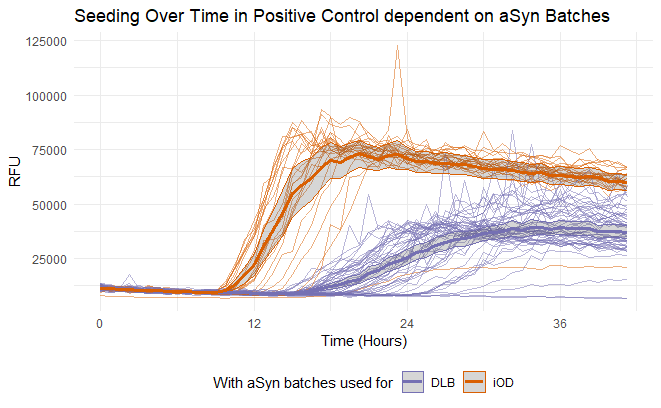
**A B**

*A: Time plot of the seed amplification in positive controls (brain homogenate from a DLB patient) run with different recombinant α-synuclein batches. Bath 1 was used for iOD/HC samples and Bath 1+2 (with similar kinetics) were used for DLB. The positive control sample was produced uniformly from the same brain and used fresh. B: Kinetic parameters for positive control in batches used for DLB and iOD/HC.
Abbreviations: AUC = area under the curve, DLB = Dementia with Lewy bodies, Fmax = maximal fluorescence measured, HC = healthy controls, iOD = idiopathic olfactory dysfunction, IQR = interquartile range, RFU = relative fluorescence unit, TH50 = time to 50 of Fmax, Vmax = maximal velocity of RFU change over 1.5 hours.*

## Figure S4 Correlations of Symptoms


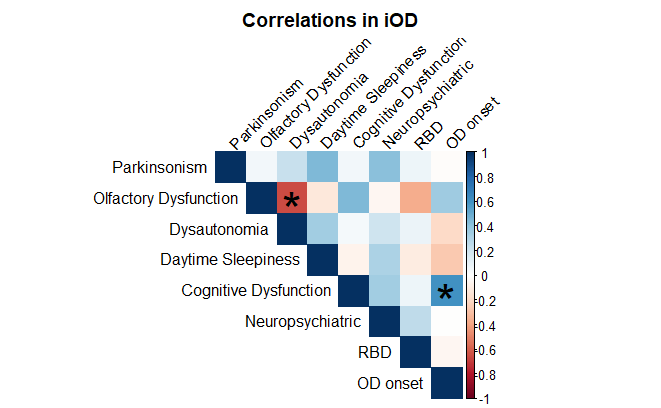

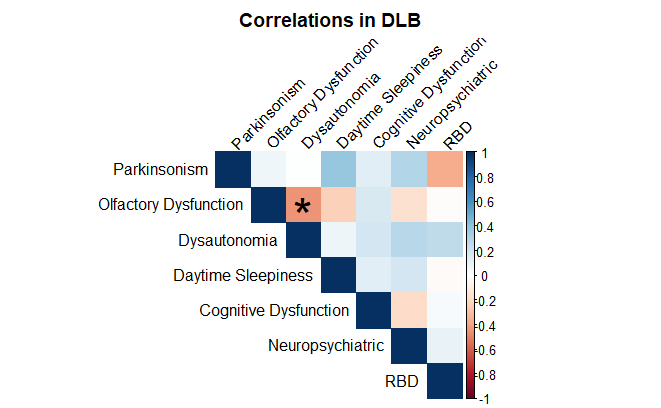

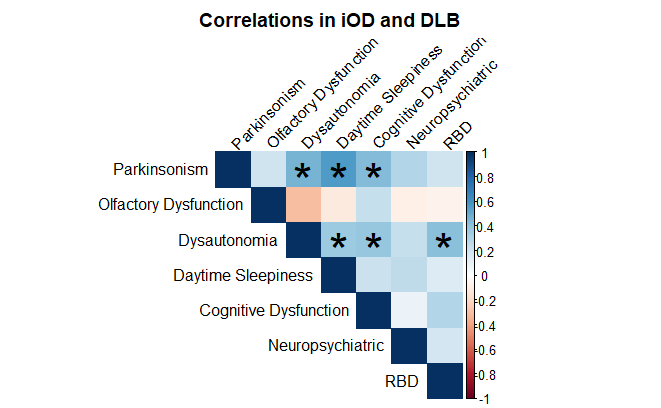


*Heatmap of Spearman's rank correlation coefficient performed on the groups: iOD, DLB, iOD, and DLB combined. The color intensity is the correlation, and * is marked on significant results (p<0.05). The correlation between olfactory dysfunction and dysautonomia in the combined iOD and DLB group has a p-value of 0.050. The following variables were used: Parkinsonism is based on MDS-UPDRS III. Olfactory dysfunction based on the inverse score of the Sniffin’ Stick Identification test (so a higher score = more dysfunction). Dysautonomia is based on the sum of values from MDS-UPDRS questions on urinary problems, constipation, lightheadedness on standing, and drooling. The neuropsychiatric score is based on the sum of values from the MDS-UPDRS questions on depression, anxiety, and apathy. Daytime Sleepiness is based on the score of MDS-UPDRS question on daytime sleepiness. Cognitive dysfunction is based on the inverse score of MoCA (so a higher score = more dysfunction). RBD is based on the RBD1Q test.*

## Figure S5 Seeding over Time in Skin with individual datapoints


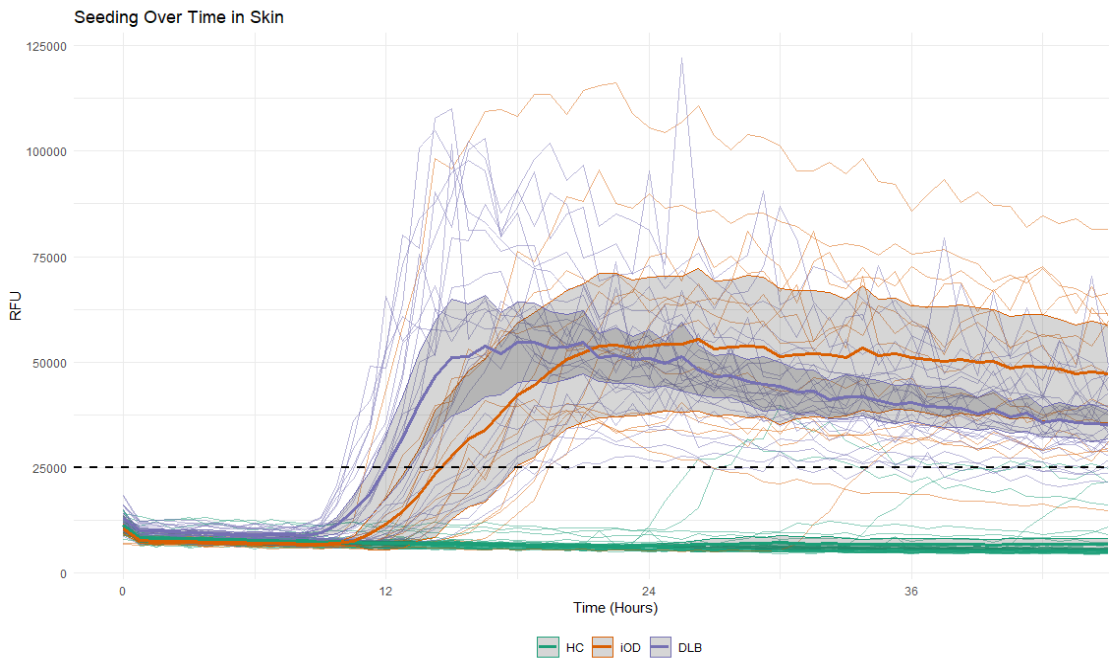


*Time plot with individual datapoints of the seed amplification from skin sample analysis with mean and CI for each group: HC (N=48), iOD (N=13) (αSyn positive in skin), and DLB (N=24)(αSyn positive in skin). The plot is based on the mean values from the two best-performing wells out of four. SAA in the skin from HC (N=48), iOD (N=13) (αSyn positive in skin), and DLB (N=24) (αSyn positive in skin) based on the mean of the two best-performing wells out of four. The dotted line represents the threshold.
Abbreviations: DLB = Dementia with Lewy bodies, HC = healthy controls, iOD = idiopathic olfactory dysfunction, RFU = relative fluorescence unit*

## Table S1 Kinetic Parameters for the α-Synuclein Seeding in Skin Samples

|  | RBD- (N=16) | RBD+ (N=21) | p-value |
| --- | --- | --- | --- |
| Time-to-threshold, median, (IQR), hours | 16 (14–19) | 13 (11–17) | 0.13 |
| TH50, median (IQR) hours | 15 (14–19) | 14 (13–17) | 0.15 |
| Vmax, median (IQR)×10³ RFU | 11 (7.4–17) | 16 (9.9–25) | 0.38 |
| Fmax, median (IQR)×10³ RFU | 61 (40–77) | 65 (58–96) | 0.26 |
| AUC, median (IQR)×10⁶ | 1.5 (0.99–1.9) | 1.6 (1.5–2.1) | 0.91 |

*Kinetic parameters for skin SAA in SAA-positive iOD and DLB with and without positive RBD1Q. See also Supplementary Materials Fig. S1 for illustrated definitions of kinetic parameters.
Abbreviations: AUC = area under the curve, DLB = Dementia with Lewy bodies, Fmax = maximal fluorescence measured, HC = healthy controls, iOD = idiopathic olfactory dysfunction, IQR = interquartile range, RFU = relative fluorescence unit, TH50 = time to 50 of Fmax, Vmax = maximal velocity of RFU change over 1.5 hours*

## Figure S6 ROC Curves for Kinetic Parameters in iOD vs DLB


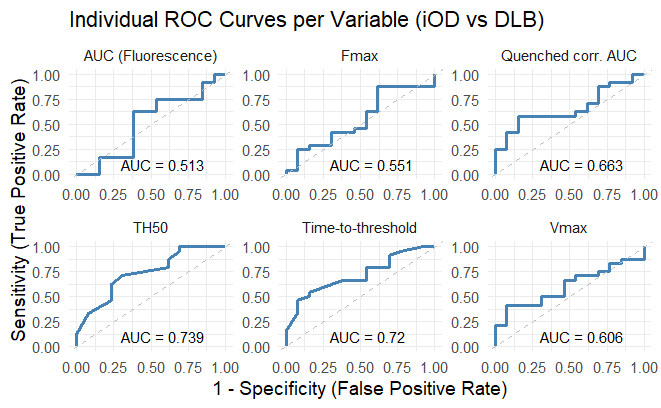


*ROC curves for the discrimination of DLB (case) from iOD by kinetic αSyn-SAA parameters. The Quenched corrected AUC = AUC x the quenching ratio. Abbreviations: AUC = area under the curve, DLB = Dementia with Lewy bodies, Fmax = maximal fluorescence measured, HC = healthy controls, iOD = idiopathic olfactory dysfunction, IQR = interquartile range, RFU = relative fluorescence unit, TH50 = time to 50 of Fmax, Vmax = maximal velocity of RFU change over 1.5 hours.*

## Table S2 The association between clinical assessments and kinetic parameters for skin SAA in iOD and DLB without correction

| Response | Predictor | β Estimate | 95% CI | p-value | R² |
| --- | --- | --- | --- | --- | --- |
| MoCA (reversed) | TH50 | -1.285 | [-2.745, 0.175] | 0.0826 | 0.084 |
|  | AUC | -0.814 | [-2.314, 0.685] | 0.278 | 0.034 |
|  | Fmax | -0.622 | [-2.132, 0.888] | 0.408 | 0.02 |
|  | Vmax | -0.164 | [-1.688, 1.360] | 0.829 | 0.001 |
|  | Time-to-threshold | -0.951 | [-2.440, 0.539] | 0.204 | 0.046 |
| Olfactory-Identification (reversed) | TH50 | 0.307 | [-0.499, 1.112] | 0.444 | 0.017 |
|  | AUC | -0.599 | [-1.385, 0.187] | 0.131 | 0.064 |
|  | Fmax | -0.548 | [-1.339, 0.242] | 0.168 | 0.054 |
|  | Vmax | -0.282 | [-1.089, 0.525] | 0.483 | 0.014 |
|  | Time-to-threshold | 0.407 | [-0.393, 1.208] | 0.309 | 0.03 |
| MDS-UPDRS I | **TH50** | **-2.155** | **[-4.035, -0.274]** | **0.026** | **0.134** |
|  | **AUC** | **2.113** | **[0.227, 3.999]** | **0.029** | **0.129** |
|  | **Fmax** | **1.934** | **[0.025, 3.842]** | **0.047** | **0.108** |
|  | **Vmax** | **2.054** | **[0.160, 3.948]** | **0.034** | **0.122** |
|  | **Time-to-threshold** | **-2.427** | **[-4.268, -0.586]** | **0.011** | **0.170** |
| MDS-UPDRS II | **TH50** | **-2.528** | **[-4.350, -0.706]** | **0.0079** | **0.185** |
|  | AUC | 0.266 | [-1.750, 2.282] | 0.790 | 0.002 |
|  | Fmax | 0.536 | [-1.473, 2.546] | 0.592 | 0.008 |
|  | Vmax | 1.581 | [-0.363, 3.525] | 0.108 | 0.072 |
|  | **Time-to-threshold** | **-2.182** | **[-4.056, -0.308]** | **0.024** | **0.138** |
| MDS-UPDRS III | **TH50** | **-2.528** | **[-9.588, -1.271]** | **0.012** | **0.167** |
|  | AUC | -1.470 | [-5.999, 3.059] | 0.514 | 0.012 |
|  | Fmax | -0.31 | [-4.866, 4.246] | 0.891 | 0.001 |
|  | Vmax | 2.665 | [-1.799, 7.129] | 0.234 | 0.04 |
|  | Time-to-threshold | -4.292 | [-8.604, 0.020] | 0.051 | 0.104 |
| MDS-UPDRS I-III | **TH50** | **-5.429** | **[-9.588, -1.271]** | **0.0120** | **0.167** |
|  | AUC | 0.951 | [-6.275, 8.178] | 0.791 | 0.002 |
|  | Fmax | 2.190 | [-5.005, 9.385] | 0.541 | 0.011 |
|  | Vmax | 6.305 | [-0.597, 13.208] | 0.072 | 0.089 |
|  | **Time-to-threshold** | **-8.906** | **[-15.463, -2.349]** | **0.009** | **0.178** |
| Positive Symptom Count | **TH50** | **-5.429** | **[-9.588, -1.271]** | **0.0120** | **0.167** |
|  | AUC | 0.402 | [-0.215, 1.018] | 0.195 | 0.048 |
|  | Fmax | 0.495 | [-0.114, 1.103] | 0.108 | 0.072 |
|  | **Vmax** | **0.912** | **[0.363, 1.461]** | **0.002** | **0.246** |
|  | **Time-to-threshold** | **-1.044** | **[-1.564, -0.524]** | **<0.001** | **0.322** |

*Table of the association between standardized clinical assessments and kinetic parameters for skin SAA in iOD and DLB, with a positive αSyn-SAA in skin. Significant predictors are in bold. Table S3 displays values corrected for age and sex.
Abbreviations: AUC = Area under the curve, CI = 95% confidence interval, Fmax = maximal fluorescence measured, MDS-UPDRS = Movement Disorders Society Unified Parkinson’s Rating Scale, MoCA = Montreal Cognitive Assessment, TH50 = time to 50 of Fmax, Vmax = maximal velocity of fluorescent change over 1.5 hours*

## Table S3 The Association between clinical assessments and kinetic parameters for skin SAA in iOD and DLB corrected for sex and age

| Response | Predictor | β Estimate | 95% CI | p-value | R² |
| --- | --- | --- | --- | --- | --- |
| MoCA (reversed) | TH50 | -0.404 | -1.833 to 1.025 | 0.569 | 0.301 |
|  | AUC | -0.744 | -2.069 to 0.581 | 0.261 | 0.321 |
|  | Fmax | -0.759 | -2.062 to 0.545 | 0.245 | 0.323 |
|  | Vmax | -0.934 | -2.274 to 0.406 | 0.166 | 0.335 |
|  | Time-to-threshold | -0.133 | -1.541 to 1.275 | 0.849 | 0.295 |
| Olfactory-Identification (reversed) | TH50 | 0.546 | -0.330 to 1.422 | 0.214 | 0.076 |
|  | AUC | -0.634 | -1.447 to 0.179 | 0.122 | 0.100 |
|  | Fmax | -0.59 | -1.394 to 0.214 | 0.145 | 0.093 |
|  | Vmax | -0.434 | -1.282 to 0.414 | 0.306 | 0.062 |
|  | Time-to-threshold | 0.621 | -0.230 to 1.473 | 0.147 | 0.092 |
| MDS-UPDRS I | TH50 | -1.637 | -3.684 to 0.409 | 0.113 | 0.184 |
|  | **AUC** | **2.404** | **0.594 to 4.214** | **0.011** | **0.279** |
|  | **Fmax** | **1.912** | **0.061 to 3.763** | **0.043** | **0.223** |
|  | Vmax | 1.631 | -0.330 to 3.592 | 0.100 | 0.189 |
|  | **Time-to-threshold** | **-1.995** | **-3.958 to -0.032** | **0.047** | **0.220** |
| MDS-UPDRS II | TH50 | -1.738 | -3.495 to 0.018 | 0.052 | 0.397 |
|  | AUC | 0.815 | -0.911 to 2.541 | 0.344 | 0.342 |
|  | Fmax | 0.61 | -1.101 to 2.321 | 0.473 | 0.334 |
|  | Vmax | 0.905 | -0.855 to 2.664 | 0.303 | 0.345 |
|  | Time-to-threshold | -1.450 | -3.202 to 0.302 | 0.102 | 0.377 |
| MDS-UPDRS III | TH50 | -3.087 | -6.881 to 0.708 | 0.107 | 0.449 |
|  | AUC | -0.286 | -3.998 to 3.426 | 0.876 | 0.403 |
|  | Fmax | -0.228 | -3.886 to 3.429 | 0.900 | 0.403 |
|  | Vmax | 0.71 | -3.076 to 4.496 | 0.705 | 0.405 |
|  | Time-to-threshold | -2.120 | -5.920 to 1.680 | 0.265 | 0.425 |
| MDS-UPDRS I-III | **TH50** | **-6.483** | **-12.23 to -0.739** | **0.028** | **0.499** |
|  | AUC | 2.966 | -2.754 to 8.686 | 0.299 | 0.438 |
|  | Fmax | 2.323 | -3.349 to 7.994 | 0.411 | 0.431 |
|  | Vmax | 3.269 | -2.561 to 9.099 | 0.262 | 0.441 |
|  | Time-to-threshold | -5.593 | -11.327 to 0.142 | 0.056 | 0.481 |
| Positive Symptom Count | **TH50** | **-0.751** | **-1.197 to -0.306** | **0.002** | **0.605** |
|  | **AUC** | **0.589** | **0.148 to 1.030** | **0.010** | **0.562** |
|  | **Fmax** | **0.502** | **0.056 to 0.949** | **0.029** | **0.537** |
|  | **Vmax** | **0.652** | **0.210 to 1.093** | **0.005** | **0.579** |
|  | **Time-to-threshold** | **-0.756** | **-1.189 to -0.323** | **0.001** | **0.612** |

*Table of the association between standardized clinical assessments and kinetic parameters for skin SAA in iOD and DLB. Significant predictors are in bold and displayed in Figure 3.
Abbreviations: AUC = Area under the curve, CI = 95% confidence interval, Fmax = maximal fluorescence measured, MDS-UPDRS = Movement Disorders Society Unified Parkinson’s Rating Scale, MoCA = Montreal Cognitive Assessment, TH50 = time to 50 of Fmax, Vmax = maximal velocity of fluorescent change over 1.5 hours.*
